# Using Compact Coevolutionary Algorithm for Matching Biomedical Ontologies

**DOI:** 10.1155/2018/2309587

**Published:** 2018-10-08

**Authors:** Xingsi Xue, Jie Chen, Junfeng Chen, Dongxu Chen

**Affiliations:** ^1^College of Information Science and Engineering, Fujian University of Technology, Fuzhou 350118, China; ^2^College of IOT Engineering, Hohai University, Nanjing 213022, China; ^3^Fujian Medical University Union Hospital, Fuzhou 350001, China

## Abstract

Over the recent years, ontologies are widely used in various domains such as medical records annotation, medical knowledge representation and sharing, clinical guideline management, and medical decision-making. To implement the cooperation between intelligent applications based on biomedical ontologies, it is crucial to establish correspondences between the heterogeneous biomedical concepts in different ontologies, which is so-called biomedical ontology matching. Although Evolutionary algorithms (EAs) are one of the state-of-the-art methodologies to match the heterogeneous ontologies, huge memory consumption, long runtime, and the bias improvement of the solutions hamper them from efficiently matching biomedical ontologies. To overcome these shortcomings, we propose a compact CoEvolutionary Algorithm to efficiently match the biomedical ontologies. Particularly, a compact EA with local search strategy is able to save the memory consumption and runtime, and three subswarms with different optimal objectives can help one another to avoid the solution's bias improvement. In the experiment, two famous testing cases provided by Ontology Alignment Evaluation Initiative (OAEI 2017), i.e. anatomy track and large biomed track, are utilized to test our approach's performance. The experimental results show the effectiveness of our proposal.

## 1. Introduction

Ontologies provide a shared and common vocabulary for representing a domain of knowledge [1]. Over the recent years, ontologies are widely used in various domains such as medical records annotation [2], medical knowledge representation and sharing, clinical guidelines management [3], and medical decision-making [4]. However, most biomedical ontologies are developed independently by different experts who might define one entity with different names or in different ways, causing the problem of ontology heterogeneity. For example, to describe the muscles that surround and power the human heart, the National Cancer Institute's thesaurus and ontology (NCI) [5] use the name “Myocardium,” whereas the Foundation Model of Anatomy (FMA) [6] uses “Cardiac Muscle Tissue.” To implement the cooperation between intelligent applications based on biomedical ontologies, it is crucial to establish correspondences between the heterogeneous biomedical concepts in different ontologies, which is so-called biomedical ontology matching.

Recently, Evolutionary Algorithms (EAs) are one of the state-of-the-art methodologies to match the heterogeneous ontologies [7]. However, huge memory consumption, long runtime, and the bias improvement of the solutions hamper EA-based ontology matching techniques from efficiently matching biomedical ontologies. Thus, besides the quality of alignments, main memory consumption and runtime needed by the ontology matcher are of prime importance when matching the biomedical ontologies. In this paper, we propose to use the compact EA [8], which utilizes a probabilistic representation of the population, to save the memory consumption of classic EA. Then, we introduce the local search strategy into its evolving process to balance the exploration and exploitation and reduce the runtime needed. On this basis, we further propose a compact Coevolutionary Algorithm, which utilizes three subswarms with different objectives to help one another to avoid the solution's bias improvement caused by traditional metric f-measure [9].

The rest of the paper is organized as follows: Section 2 describes the related works; Section 3 gives some basic concepts of ontology, ontology alignment, and the similarity measures; Section 4 presents the optimal model problem and the details of the compact Coevolutionary Algorithm for matching biomedical ontologies; Section 5 gives the experimental results and relevant analysis; finally, Section 6 draws the conclusions.

## 2. Related Work

### 2.1. Evolutionary Algorithm-Based Ontology Matching Technique

Due to the complex and time-consuming nature of the ontology matching process, EA-based methods could present a good methodology for obtaining ontology alignments and indeed have already been applied to solve the ontology alignment problem by reaching acceptable results [10]. Different from other EA based approaches [11–13] which models the ontology alignment process as a meta-matching problem, i.e. how to determine the best appropriate weight configuration in ontology matching process in order to obtain a satisfactory alignment, in this work, ontology matching problem is considered as a global entity matching problem. Genetic Algorithm-Based Ontology Matching (GAOM) [14] is the representative system, which utilized Genetic Algorithm (GA) to determine the optimal ontology alignment. Particularly, GAOM utilizes the chromosomes to describe the potential alignments between two ontologies and utilizes GAs to determine the optimal solution. Besides, MapPSO and MapEVO [15] which exploited the Particle Swarm Optimization Algorithm (PSO) [16] and Evolutionary Programming (EP) [17], respectively, also adopted this idea. Acampora et al. [18] designed a Memetic Algorithm (MA) which introduced a local search process to improve the performance of EA. More recently, Xue et al. [19, 20], respectively, used the compact EA and compact Population-Based Incremental Learning Algorithm (PBIL) to save the memory consumption without sacrificing the solution's quality. Compact EA and compact PBIL represented the population as a probability vector (PV) over the set of solutions and are operationally equivalent to the order-one behaviour of the simple EA with uniform crossover. In this way, a much smaller number of solutions must be stored in the memory, thus significantly reducing the memory consumption.

### 2.2. Coevolutionary Algorithm

The Coevolutionary Algorithm [21] makes multiple swarms simultaneously evolve and communicate with one another to improve the search performance. Currently, distributed coevolution is the most popular coevolving process, which shares the search information among multiple swarms through the population migration strategy. During the searching process, different swarms have evolving strategies and configurations. Tan et al. [22] proposed to decompose the problem's solution vector into multiple swarms to evolve simultaneously. Mu and Liu [23] presented an M-elite Coevolutionary Algorithm that applied different elite strategies in the coevolving process. The elite centered swarm has the highest priority, and other swarms implemented the cooperative coevolving process. In [24], a parallel evolving mechanism was designed by dividing the population into three swarms that evolved independently. However, all the swarms use the same evolving strategy, and the swarm's evolving process swarm was relatively independent, which decreased the algorithm's exploration and exploitation ability. More recently, Wang et al. [25] proposed a two-elite strategy which makes use of the differences between two elites to guide the whole evolving process.

Different from all the techniques mentioned above, in this work, we propose a compact coevolutionary Algorithm to match the biomedical ontologies, which combines the advantages of the compact EA and coEvolutionary Algorithm to save the memory consumption and runtime and overcome the bias improvement of solutions.

### 2.3. Preliminaries

#### 2.3.1. Ontology, Ontology Alignment, and Ontology Matching Process

In this work, an ontology is defined as a quadruple *O*=(*C*, *P*, *I*, *A*), where

- *C* is the class set, i.e., the set of concepts that populate the domain of interest,
- *P* is the property set, i.e., the set of relations between the concepts of domain,
- *I* is the instance set, i.e., the set of objects in the real world representing the instances of a concept, and
- *A* is the axiom set, i.e., the statements that say what is true about the modeled domain.

An alignment A between two ontologies *O*_1_ and *O*_2_ is defined as a set of correspondences, and each correspondence is a triple (*e*_1_, *e*_2_, *n*), where *e*_1_ and *e*_2_ are the entities in *O*_1_ and *O*_2_, respectively, and *n* ∈ [0,1] is a confidence value holding for the correspondence between them. In this work, the relation existing between two ontology entities is the equivalence (=). The ontology matching process can be defined as a function *θ*(*O*_1_, *O*_2_, *p*, *r*) [26], where *p* is the parameter set and *r* is the resource set. Ontology matching process returns a new alignment *A*_*N*_ between ontologies *O*_1_ and *O*_2_.

#### 2.3.2. Concept Similarity Measure

Concept similarity measure is the foundation of biomedical ontology matching [27]. In this work, we utilize an asymmetrical concept similarity measure to calculate the biomedical concepts' similarity values. First, for each biomedical concept, we construct a profile for it by collecting the label, comment, and property information such as label, domain, and range, from itself and all its direct descendants. Then, the similarity of two biomedical concepts *c*_1_ and *c*_2_ is measured based on the similarity of their profiles *p*_1_ and *p*_2_, which can be calculated by the following two asymmetrical measures:

(1) $$\begin{array}{c} sim_{1}\left(p_{1},p_{2}\right)=\frac{\left|p_{1}\cap p_{2}\right|}{p_{1}},\\ sim_{2}\left(p_{1},p_{2}\right)=\frac{\left|p_{1}\cap p_{2}\right|}{p_{2}}, \end{array}$$

where |*p*_1_| and |*p*_2_| are the cardinalities of the profile *p*_1_ and *p*_2_, respectively, |*p*_1_∩*p*_2_| is the number of identical elements in *p*_1_ and *p*_2_. The similarity value of *e*_1_ and *e*_2_ is equal to (*sim*_1_(*p*_1_, *p*_2_)+*sim*_2_(*p*_1_, *p*_2_))/2 when |*sim*_1_(*p*_1_, *p*_2_) − *sim*_2_(*p*_1_, *p*_2_)| ≤ *δ*, and otherwise, 0.

In this work, *δ* is the threshold to measure the extent of the semantic equivalence between *sim*_1_(*p*_1_, *p*_2_) and *sim*_2_(*p*_1_, *p*_2_). When the similarity value between two profile elements is above the threshold, they are identified as semantically similar. Generally, *δ* should be set relatively small to reflect *sim*_1_(*e*_1_, *e*_2_) and *sim*_2_(*e*_1_, *e*_2_) have little difference when the entity *e*_1_ and *e*_2_ are semantically equivalent. However, if *δ* is too small, we would miss many semantically equivalent terms. Therefore, the suggested domain of *δ* is [0.01, 0.10]. In this work, to obtain a suitable, we conducted a pre-experiment on the benchmark by varying the value of *δ* in its suggested domain, and found the semantic equivalence performs well when *δ* is assigned to 0.06.

Moreover, the similarity value of two profile elements is calculated by N-gram distance [28], which is the most performing string-based similarity measure for the biological ontology matching problem, and a linguistic measure, which calculate a synonymy-based distance through the Unified Medical Language System (UMLS) [29]. Given two words *w*_1_ and *w*_2_, their similarity *sim*_2_(*w*_1_, *w*_2_) is equal to 1 when two words are synonymous, and otherwise, *N* − gram(*w*_1_, *w*_2_).

### 2.4. Compact Coevolutionary Algorithm

#### 2.4.1. Rough Alignment Evaluations

In this work, we suppose that, in the golden alignment, one concept in the ontology is matched with only one concept in the other ontologies and vice versa. Two rough alignment evaluations, i.e., *MatchCoverage* and *MatchRatio*, are utilized to measure the alignment's quality. In particular, *MatchCoverage* is utilized to approximate recall [9], which calculates the fraction of concepts which exist in at least one correspondence in the resulting alignment in comparison to the total number of concepts in the ontology. The formula of it is presented as follows:

(2) $$\begin{array}{c} MatchCoverage=\frac{\left|C_{O_{1}-Match}\right|+\left|C_{O_{2}-Match}\right|}{\left|C_{O_{1}}\right|+\left|C_{O_{2}}\right|}\epsilon \left[0,1\right], \end{array}$$

where

- *C*_*O*_1_−*Match*_ and *C*_*O*_2_−*Match*_ are the matched concept sets of ontology *O*_1_ and *O*_2_, respectively; and
- *C*_*O*_1__ and *C*_*O*_2__ are the concept sets of ontology *O*_1_ and *O*_2_, respectively.

And, *MatchRatio* is used to approximate precision [9], which calculates the ratio between the number of found correspondences and the number of matched concepts. The formula of it is presented as follows:

(3) $$\begin{array}{c} MatchRatio=\frac{\left|C_{O_{1}-Match}\right|+\left|C_{O_{2}-Match}\right|}{2\cdot \left|Corr_{O_{1}-O_{2}}\right|}\epsilon \left[0,1\right], \end{array}$$

where

- *Corr*_*O*_1_−*O*_2__ is the correspondence set in the alignment; and
- *C*_*O*_1_−*Match*_ and *C*_*O*_2_−*Match*_ are the matched concept sets of ontology *O*_1_ and *O*_2_, respectively;

In most instances, it requires considering both *MatchCoverage* and *MatchRatio* to measure the alignment's quality. By referring to the most common combining function f-measure [9], we define *MatchFmeasure* as follows:

(4) $$\begin{array}{c} MatchFmeasure=2\times \frac{MatchCoverage\cdot MatchRatio}{MatchCoverage+MatchRatio}. \end{array}$$

#### 2.4.2. The Optimal Model for Ontology Entity Matching Problem

Given two biomedical ontologies *O*_1_ and *O*_2_, we take maximizing *MatchFmeasure* as the goal, and the optimal model for ontology entity matching problem can be defined as follows:

(6) $$\begin{array}{c} \max\;MatchFmeasure\left(X\right),\\ \text{s.t.}\;X={\left(x_{1},x_{2},\ldots ,x_{\left|O_{1}\right|},x_{\left|O_{1}\right|+1},\right)}^{T},\\ x_{i}=1,2,\ldots ,x_{\left|O_{2}\right|},\\ x_{\left|O_{1}\right|+1}\in \left[0,1\right], \end{array}$$

where the decision variable *X* represents an alignment between *O*_1_ and *O*_2_, *x*_*i*_ represents the *i*th correspondence between *i*th concept in *O*_1_ and *x*_*i*_th concept in *O*_2_, |*O*_1_| and |*O*_2_| are the cardinalities of the concept set in *O*_1_ and *O*_2_, respectively, and *x*_|*O*_1_|+1_ ∈ [0,1] is the threshold to filter the final alignment.

One of the shortcomings of *MatchFmeasure* is that the improvement of it does not say anything about whether both *MatchCoverage* and *MatchRatio* are simultaneously improved or not. In other words, no matter how large a measured improvement in *MatchFmeasure* is, it can still be extremely dependent on the improvement on one of the individual metrics [30]. To overcome this bias improvement, we propose a compact coevolutionary Algorithm, which has three PVs that characterize subswarms that aim at maximizing *MatchCoverage*, *MatchRatio*, and *MatchFmeasure*, respectively. Through the cooperation of three PVs, we dedicate to ensure the simultaneous improvement on *MatchCoverage* and *MatchRatio* during the evolving process.

#### 2.4.3. Compact Evolutionary Algorithm

Model-based optimization using probabilistic modeling of the search space is one of the areas where research on Compact Evolutionary Algorithm (CEA) has considerably advanced in recent years. In each generation, CEA updates the probability vector (PV), which is a probabilistic model describing the univariate statistics of the best solutions and then uses it to generate new candidate solutions. By employing the PV, instead of a population of solutions, to simulate the behavior of classic EA, a much smaller number of individuals is needed to be stored in the memory. Thus, CEA can significantly reduce the memory consumption [31]. In order to further improve CEA performance, we introduce the local search strategy into CEA's evolving process. This marriage between global search and local search is helpful in reducing the possibility of the premature convergence and increasing the convergence speed.

In the next, three main components of CEA, i.e., chromosome-encoding mechanism, probability vector, and local search strategy are, respectively, presented.

1. *Chromosome-Encoding Mechanism*: in this work, the genes are encoded through the binary coding mechanism and can be divided into two parts. The first part stands for the correspondences in the alignment, and the other one stands for a threshold. Given the total number *n*_1_ and *n*_2_ of two biomedical concepts in ontologies, the first part of a chromosome (or PV) consists of *n*_1_ gene segments, and the binary code length (BCL) of each gene segment is equal to log_2_(*n*_2_)+0.5, which ensures each gene segment could present any target ontology class's index, while the second part of a chromosome (or PV) has only one gene segment, whose BCL is equal to log_2_(1/*numAccuracy*)+0.5, which can ensure this gene segment could present any threshold value under the numerical accuracy *numAccuracy*. Thus, the total length of the chromosome (or PV) is equal to *n*_1_ × log_2_(*n*_2_)+0.5+log_2_(1/*numAccuracy*)+0.5.

Given a gene segment *geneSeg*={*geneBit*_1_, *geneBit*_2_, ⋯*geneBit*_*n*_, }, where *geneBit*_*i*_ is the *i*th gene bit value of the gene segment, we decode to obtain a decimal number whose value is equal to ∑_*i*=1_^*n*^2^*geneBit*_*i*_^. In particular, with respect to the first part decoding results, the decimal numbers obtained represent the indexes of the target classes, where 0 means the source instance is not mapped to any target ontology's class. With regard to the second part of decoding result, the decimal number obtained should multiply the threshold's numerical accuracy. Last but not least, if a decimal number *d* obtained is larger than *u*, we will replace it with *u*/*d*.

(2) *Probability Vector*: in general, CEA aims at generating a PV which represents a population of high evaluation solutions, and its operations take place directly on the PV. In this work, the number of elements in PV is equal to the number of individual's gene bits and each element's value is in [0,1], and here is an example on how to use PV (0.5, 0.9, 0.3, 0.8)^*T*^ to generate a new solution. First, generate four random numbers, such as 0.6, 0.5, 0.8, and 0.9. Then, compare the numbers with the elements in PV accordingly to determine the new generated individual's gene values. For example, since 0.6 > 0.5, the first gene bit's value of the new solution is 0, and similarly, the remaining gene bits' values are 1, 0, and 0, respectively. In this way, the new solution we obtain is 0100. By repeating this procedure, we can obtain various individuals. In addition, if 0100 is the elite solution in the current generation, PV should be updated according to its information. Given PV's update rate, say 0.1, if the gene value of the elite is 0, the corresponding element of PV will minus 0.1, otherwise add 0.1. In this way, the updated PV is (0.4, 1.0, 0.2, 0.7)^*T*^.
(3) *Local Search Strategy*: local search process tries to improve the elite solution by searching in the neighborhood of it. In this work, we utilize a crossover operator to implement the local search process, which randomly copies a sequential fragment of *ind*_*new*_'s genes into the corresponding positions of *ind*_*neighbor*_, to generate a new solution. For the sake of clarity, given the length of the chromosome *len* and the crossover probability *p*_*c*_, the pseudocode of the binary crossover operator is shown in Algorithm 1.

This procedure is similar with the two-point crossover where the first cut point is randomly selected from {1; 2; ···; *len*}, and the second point is determined such that *L* consecutive genes (counted in a circular manner) are taken from *ind*_*new*_. Since *ind*_*new*_ and *ind*_*elite*_ are both generated through the PV, most of their gene bit values are the same. Therefore, even when *p*_*c*_ is large, *ind*_*neighbor*_ only mutates a few gene bit values of *ind*_*elite*_. In this sense, this variation operator can be considered fairly exploitative.

#### 2.4.4. Pseudocode of Compact Coevolutionary Algorithm

In this work, we use three PVs to represent the subswarms for maximizing *MatchRatio*, *MatchCoverage*, and *MatchFmeasure*, respectively. In particular, the PV here represents the population that consists of the solutions of its corresponding representative subproblem and this problem's neighbor subproblems. Finally, these PVs help each other in the process of determining three representative solutions, which are given in the following. Here, we mark three representative subproblems of maximizing *MatchRatio*, maximizing *MatchCoverage*, and maximizing *MatchFmeasure* with the symbols *P*_*mr*_, *P*_*mc*_, and *P*_*mf*_, respectively, and three PVs for solving *P*_*mr*_, *P*_*mc*_, and *P*_*mf*_ with the symbols *PV*_*mr*_, *PV*_*mc*_, and *PV*_*mf*_, respectively. We present the pseudocode of compact Coevolutionary Algorithm in Algorithm 2.

### 2.5. Experimental Results and Analysis

In this work, we exploit the Anatomy (http://oaei.ontologymatching.org/2017/anatomy/index.html) and Large Biomed (http://www.cs.ox.ac.uk/isg/projects/SEALS/oaei/2017/) track to study the effectiveness of our approach, which are provided by the Ontology Alignment Evaluation Initiative (OAEI 2017) (http://oaei.ontologymatching.org/2017). The Anatomy track includes two ontologies (1 task), i.e., the Adult Mouse Anatomy (AMA) ontology (2,744 classes) and a part of NCI describing the human anatomy (3,304 classes). Large Biomed track (3 tasks) aims at finding alignments between FMA, SNOMED CT, and NCI, which, respectively, contains 78,989, 122,464, and 66,724 classes. Particularly, The large Biomedic track is split into three matching problems: FMA-NCI, FMA-SNOMED, and SNOMED-NCI and each matching problem in these tasks involving different fragments of the input ontologies.

The Compact Coevolutionary Algorithm uses the following parameters which represent a trade-off setting obtained in an empirical way to achieve the highest average alignment quality on all exploited testing datasets:

- Numerical accuracy = 0.01;
- Update rate = 0.1;
- Crossover probability = 0.6;
- Mutation probability = 0.03;
- Mutation rate = 0.05;
- Maximum generation = 3000.

## 3. Results and Analysis

In order to compare the quality of our proposal with the participants of OAEI 2017 (http://oaei.ontologymatching.org/2017/results/index.html) and Population-Based Incremental Learning Algorithm (PBIL) [20], which is a state-of-the-art compact EA-based ontology matching technique, we evaluate the obtained alignments with traditional recall, precision, and f-measure. PBIL and our approach's results in Table 1 and Table 2 are the mean values in thirty time independent executions. The symbols *P*, *R*, and *F* in tables stand for precision, recall, and f-measure, respectively.

As can be seen from Table 1, our approach's f-measure outperforms all the competitors, and our approach's runtime is ranked the 4th place. In Table 2, our approach's f-measure is the highest in task1, task2, and task3. For the running time, in task1 and task 2, our approach is in the 3rd place and 4th place in task3. In both tracks, our approach outperforms AML, which is the top ontology matcher and developed primarily for the biomedical ontology matching, in all tasks in terms of f-measure, and the runtime in our approach is also very close to or less than AML. The experimental results show that the cooperation among three swarms with different objectives can effectively overcome the bias improvements and improve the quality of biomedical ontology alignments.

In particular, PBIL works with one PV, but our approach utilizes three PVs to cooperate with each other during the evolving process to improve the solution's quality. As can be seen from the experimental results, although our approach takes only a little more runtime than PBIL, the qualities of our results are much better than PBIL in terms of both recall and precision, which shows that our approach can effectively overcome the bias improvement of solutions in PBIL.

## 4. Conclusion

In this work, in order to overcome the drawbacks in traditional E-based ontology matching techniques, we for the first time propose a compact Coevolutionary Algorithm to efficiently match the biomedical ontologies. In our approach, three PVs are utilized to characterize three subswarms that take as objectives maximizing *MatchCoverage*, *MatchRatio*, and *MatchFmeasure*, respectively, and in each generation, PVs are first updated with CEA paradigm and then help each other to search for better solutions in the search space. In the experiment, OAEI 2017's Anatomy track and Large Biomed track are utilized to test our approach's performance, and the results show that our approach can efficiently determine better ontology alignments than state-of-the-art biomedical ontology matching techniques.

## Figures and Tables

**Algorithm 1:**
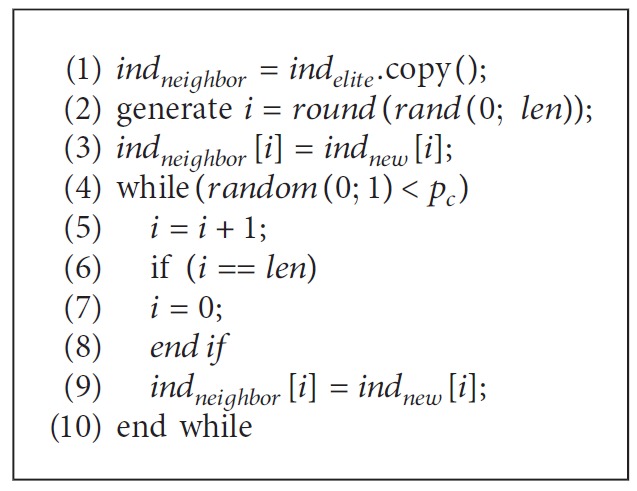


**Algorithm 2:**
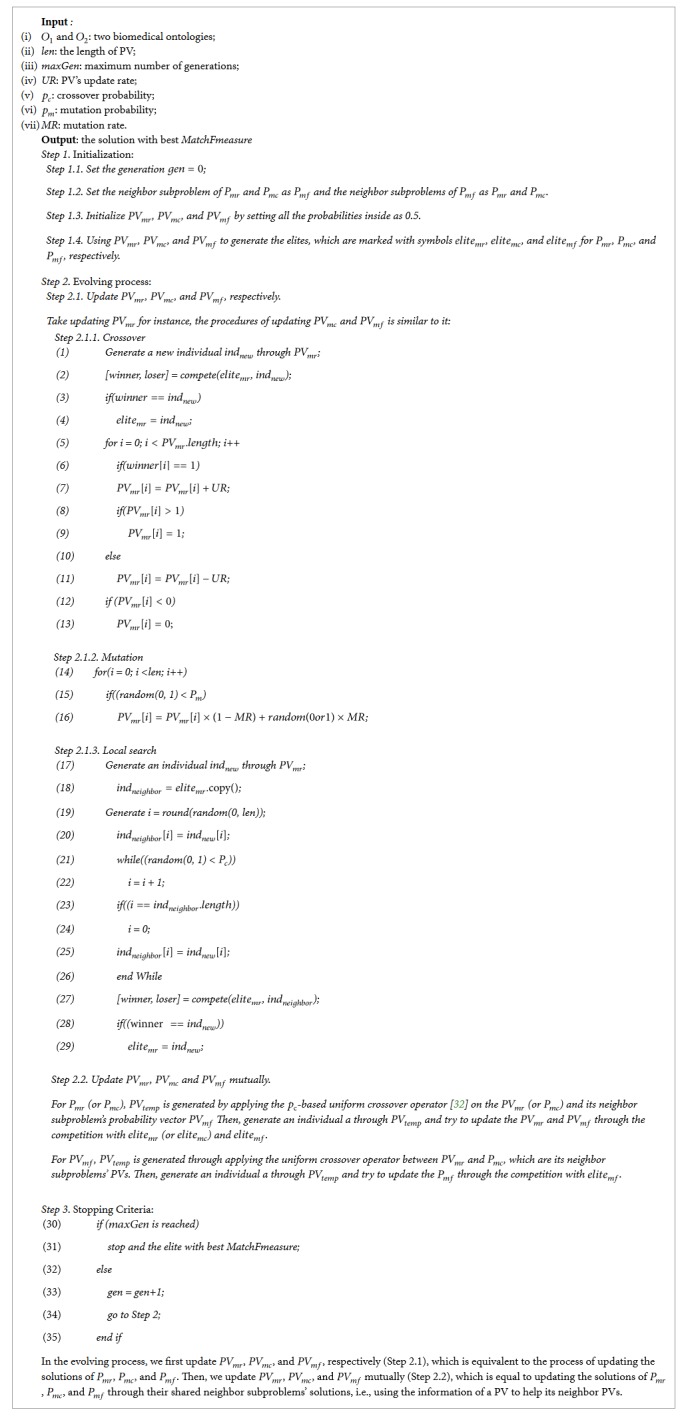


**Table 1:** Comparison of our approach with the participants in OAEI 2017 on anatomy track.

System	R	P	F	Runtime (second)
AML	0.93	0.95	0.94	37
*YAM-BIO*	0.92	0.94	0.93	70
POMap	0.90	0.94	0.93	808
LogMapBio	0.89	0.88	0.89	820
XMap	0.86	0.92	0.89	37
LogMap	0.84	0.91	0.88	22
KEPLER	0.74	0.95	0.83	234
LogMapLite	0.72	0.96	0.82	19
SANOM	0.77	0.89	0.82	295
Wiki2	0.73	0.88	0.80	2204
ALIN	0.33	0.99	0.50	836
EA	0.76	0.88	0.78	22
Our approach	0.94	0.97	0.95	34

**Table 2:** Comparison of our approach with the participants in OAEI 2017 on the Large Biomed track.

System	*R*	*P*	*F*	Runtime (second)
*Task1: whole FMA and NCI ontologies*				
XMap	0.85	0.88	0.87	130
AML	0.87	0.84	0.86	77
*YAM-BIO*	0.89	0.82	0.85	279
LogMap	0.81	0.86	0.83	92
LogMapBio	0.83	0.82	0.83	1552
LogMapLite	0.82	0.67	0.74	10
Tooll	0.74	0.69	0.71	1650
PBIL	0.76	0.88	0.78	22
Our approach	0.87	0.89	0.88	72

*Task2: whole FMA and SNOMED ontologies*				
XMap	0.84	0.77	0.81	625
YAM-BIO	0.73	0.89	0.80	468
AML	0.69	0.88	0.77	177
LogMap	0.65	0.84	0.73	477
LogMapBio	0.65	0.81	0.72	2951
LogMapLite	0.21	0.85	0.34	18
Tooll	0.13	0.87	0.23	2140
PBIL	0.72	0.74	0.72	147
Our approach	0.81	0.84	0.82	183

*Task3: whole SNOMED and NIC ontologies*				
AML	0.67	0.90	0.77	312
YAM-BIO	0.70	0.83	0.76	490
LogMapBio	0.64	0.84	0.73	4728
LogMap	0.60	0.87	0.71	652
LogMapLite	0.57	0.80	0.66	22
XMap	0.55	0.82	0.66	563
Tooll	0.22	0.81	0.34	1105
PBIL	0.64	0.81	0.71	304
Our approach	0.73	0.88	0.79	326

## Data Availability

The data used to support the findings of this study have not been made available because of the protection of technical privacy and confidentiality.
